# Advancing medical imaging: detecting polypharmacy and adverse drug effects with Graph Convolutional Networks (GCN)

**DOI:** 10.1186/s12880-024-01349-7

**Published:** 2024-07-15

**Authors:** Omer Nabeel Dara, Abdullahi Abdu Ibrahim, Tareq Abed Mohammed

**Affiliations:** 1 Collage of Engineering, Department of Electrical and Computer Engineering, Altinbas University, Istanbul, Turkey; 2 College of Computer Science and Information Technology, Department of Information Technology, University of Kirkuk, Kirkuk, Iraq

**Keywords:** Polypharmacy, Side effects, Graph Convolutional Network (GCN), Pharmacovigilance, Confusion matrix, Healthcare decision-making

## Abstract

Polypharmacy involves an individual using many medications at the same time and is a frequent healthcare technique used to treat complex medical disorders. Nevertheless, it also presents substantial risks of negative medication responses and interactions. Identifying and addressing adverse effects caused by polypharmacy is crucial to ensure patient safety and improve healthcare results. This paper introduces a new method using Graph Convolutional Networks (GCN) to identify polypharmacy side effects. Our strategy involves developing a medicine interaction graph in which edges signify drug-drug intuitive predicated on pharmacological properties and hubs symbolize drugs. GCN is a well-suited profound learning procedure for graph-based representations of social information. It can be used to anticipate the probability of medicate unfavorable impacts and to memorize important representations of sedate intuitive. Tests were conducted on a huge dataset of patients’ pharmaceutical records commented on with watched medicate unfavorable impacts in arrange to approve our strategy. Execution of the GCN show, which was prepared on a subset of this dataset, was evaluated through a disarray framework. The perplexity network shows the precision with which the show categories occasions. Our discoveries demonstrate empowering advance within the recognizable proof of antagonistic responses related with polypharmaceuticals. For cardiovascular system target drugs, GCN technique achieved an accuracy of 94.12%, precision of 86.56%, F1-Score of 88.56%, AUC of 89.74% and recall of 87.92%. For respiratory system target drugs, GCN technique achieved an accuracy of 93.38%, precision of 85.64%, F1-Score of 89.79%, AUC of 91.85% and recall of 86.35%. And for nervous system target drugs, GCN technique achieved an accuracy of 95.27%, precision of 88.36%, F1-Score of 86.49%, AUC of 88.83% and recall of 84.73%. This research provides a significant contribution to pharmacovigilance by proposing a data-driven method to detect and reduce polypharmacy side effects, thereby increasing patient safety and healthcare decision-making.

## Introduction

**Fig. 1:**
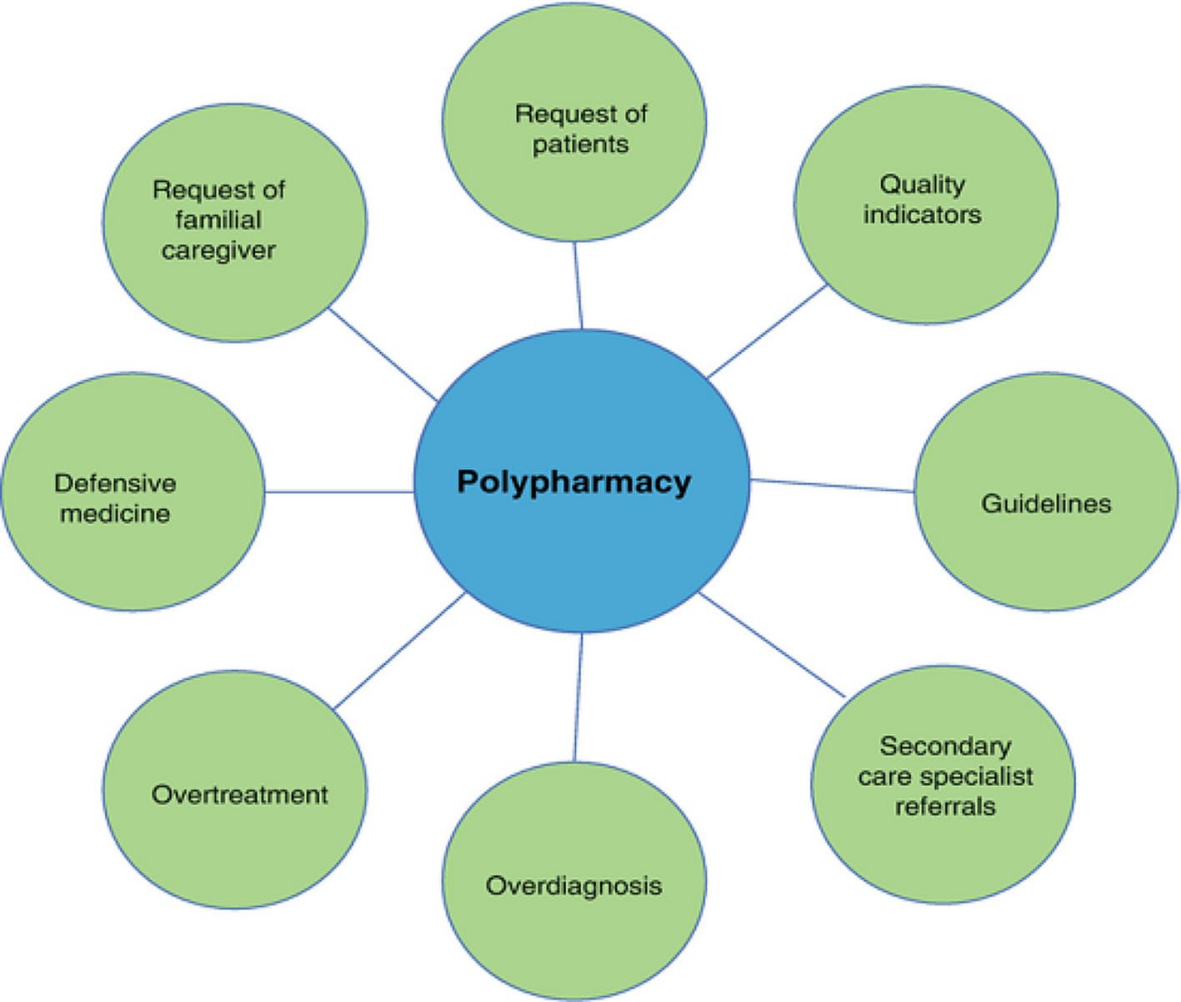
The driver of polypharmacy for treatment [6]

Polypharmacy, which alludes to the concurrent organization of different drugs by a single person, may be a broad event within the healthcare division, particularly among the elderly and those with chronic conditions [1]. The use of several pharmaceuticals and medicines can be harmful because potential dangers such as adverse drug reactions (ADRs) and drug-drug interactions (DDIs) are likely to happen. Particularly in seniors, DDIs may increase the likelihood of illness and potentially mortality. As polypharmacy becomes more sophisticated and prevalent, the understanding of its multiple facets is growing. Prior investigations have highlighted that about 30% of senior patients are utilizing five or more medicines, hence increasing the possibilities for both ADRs and DDIs [1, 2]. While the use of numerous drugs is effective in treating chronic diseases, it has been associated with side effects that require cautious supervision. In spite of the fact that polypharmacy can be a vital component within the administration of intricate restorative conditions and the upgrade of persistent results, it too postures impressive impediments within the frame of increased unfavorable sedate responses (ADRs), drug-drug intuitive (DDIs), and pharmaceutical blunders [2]. The perplexing interaction among different solutions in a patient’s treatment arrange may result in unexpected unfavorable impacts, compromised viability of treatment, and negative health results. As a result, the recognizable proof and control of antagonistic impacts related with polypharmacy have risen as basic concerns in cutting edge healthcare, as referenced in [3]. Ordinary strategies for assessing the security and viability of solutions as often as possible depend on manual pharmacovigilance methods, which are characterized by their requesting asset prerequisites, vulnerability to human blunder, and confined adaptability [4]. In addition, in circumstances including polypharmacy, conventional measurable and machine learning strategies might encounter difficulties in capturing the complex interconnects between solutions and their conceivable unfavorable impacts. In light of these deterrents, there’s an expanding slant towards utilizing modern computational strategies, such as profound learning procedures, for the reason of looking at and translating complex healthcare information. As already expressed in reference [5], Graph Convolutional Networks (GCNs) have surfaced as strong displaying instruments for social information that’s organized as graphs. By capitalizing on the inherent interconnects and reliance’s display in graph-structured information, GCNs are able of proficiently capturing perplexing designs and connections, rendering them profoundly appropriate for applications counting the expectation of sedate intelligent and the discovery of unfavorable occasions. As specified in [6], GCNs give a promising strategy for dissecting pharmaceutical interaction systems and recognizing potential unfavorable impacts related with particular drug combinations within the setting of polypharmacy as shown in Fig. 1.

Polypharmacy presents the significant challenge of characterizing adverse effects in patients taking numerous drugs with the goal of optimizing patient safety and overall health outcomes. Traditional techniques for assessing the safety and efficacy of pharmaceuticals rely heavily on human intervention in pharmacovigilance activities, which prove to be time-consuming, prone to error, and rarely scalable. These approaches are particularly unsuccessful in modeling various drug interactions and require new methods for analyzing and understanding intricate healthcare information.

In this research, we propose a novel technique for the discovery of polypharmacy side impacts utilizing GCNs. Our approach includes building a medicine interaction graph where hubs speak to person drugs, and edges indicate potential intuitive based on known pharmacological properties, sedate targets, and metabolic pathways as said in [7]. By preparing a GCN on this graph-structured information, we point to memorize important representations of sedate intuitive and foresee the probability of unfavorable effects resulting from polypharmacy. To assess the adequacy of our approach, we use a comprehensive dataset of quiet pharmaceutical records, commented on with watched side impacts and antagonistic occasions as specified in [8]. We prepare and approve our GCN show utilizing this dataset and evaluate its execution utilizing different measurements, counting precision, affectability, specificity, and region beneath the collector working characteristic curve (AUC-ROC). Moreover, we utilize perplexity framework examination to supply nitty gritty bits of knowledge into the model’s classification execution, counting genuine positives, genuine negatives, wrong positives, and untrue negatives as specified in [9]. Through us inquire about, we point to contribute to the progression of pharmacovigilance hones by presenting a data-driven approach for recognizing and moderating polypharmacy-related side impacts as said in [10]. This Paper provides a novel technique that leverages Graph Convolutional Networks (GCNs) to identify polypharmacy side effects by generating a drug interaction graph for pharmaceuticals, where the nodes are distinct drugs and the edges indicate probable interactions based on specified criteria. By applying GCNs, which are efficient at processing graph-structured data, our goal is to reproduce the linkages and dependencies to forecast the likely side effects of pharmaceuticals, thereby enhancing pharmacovigilance methods and improving patient care. This research employs datasets obtained from electronic health records (EHR) and pharmacovigilance databases to construct and verify our Graph Convolutional Network (GCN) model for forecasting polypharmacy-induced adverse effects. It is crucial to recognize that the data used in our study is prone to various biases, such as sample data imbalances and assumptions on the pharmacokinetics of drugs. The presence of these biases may compromise our capacity to apply our results to other patient groups and healthcare settings. By tackling the control of GCNs and graph-based representations of medicine intelligent, we look for to progress quiet security, optimize healthcare decision-making, and upgrade the quality of care in clinical settings.

### Problem statement

Polypharmacy, which is the concurrent organization of numerous solutions for the treatment of complex restorative conditions, could be a far reaching wonder within the healthcare industry. In spite of the fact that the implementation of polypharmacy may abdicate positive comes about for patients, it isn’t without its inalienable dangers, which contain pharmaceutical blunders, antagonistic sedate responses (ADRs), and drug-drug intelligent (DDIs). The distinguishing proof and compelling administration of potential antagonistic impacts in polypharmacy regimens show significant challenges for healthcare suppliers due to the complex nature of medicate intuitive [11]. Display strategies for assessing the security and viability of solutions regularly depend on manual pharmacovigilance methods and customary measurable examinations. These approaches are characterized by their resource-intensive nature, long execution times, and limited versatility. Besides, it has been famous in [12] that conventional machine learning strategies might encounter challenges in capturing the complex interconnects and reliance’s that exist among medicines within the setting of polypharmacy. Advanced computational procedures that can dissect complex pharmaceutical interaction systems and identify potential antagonistic impacts connected to polypharmacy regimens are direly required.

Discovery and moderation of polypharmacy-related unfavorable impacts through Graph Convolutional Network is the issue tended to in this think about. Our essential objective is to plan a data-centric strategy that can independently distinguish and categories antagonistic medicate responses and intelligent that emerge as a result of polypharmacy. Our objective is to utilize GCNs, which excel at speaking to social information within the shape of graphs, in arrange to discover critical delineations of pharmaceutical intuitive and estimate the likelihood of unfavorable impacts connected to specific medicate combinations.

The principal challenges that necessitate attention encompass:

1. Modeling Complex Drug Interactions: Polypharmacy includes the synchronous utilize of numerous drugs, driving to complex designs of sedate intuitive. Capturing these complex connections inside a computational demonstrate requires strong strategies able of dealing with graph-structured information successfully.
2. Predicting Adverse Events: Recognizing potential side impacts coming about from polypharmacy regimens may be a challenging assignment, because it requires the capacity to observe unobtrusive designs and affiliations inside large-scale pharmaceutical interaction systems as specified in [13]. Creating precise forecast models competent of identifying unfavorable occasions with tall affectability and specificity is fundamental for moving forward quiet security.
3. Scalability and Generalization: The proposed approach ought to be adaptable and generalizable to different persistent populaces and medicine regimens. Healthcare datasets change in measure, complexity, and heterogeneity, requiring strong models competent of taking care of real-world information with changing degrees of complexity.

### Aim of study

The essential point of this ponder is to contribute to the progression of pharmacovigilance hones by proposing a novel approach for the location and relief of polypharmacy-related side impacts utilizing Graph Convolutional Network (GCNs). Particularly, the research points to realize the taking after commitments:

1. Development of a Data-Driven Framework: We point to create a data-driven system leveraging GCNs to consequently recognize and classify unfavorable sedate responses (ADRs) and drug-drug intelligent (DDIs) coming about from polypharmacy regimens. By tackling the control of profound learning procedures, our system looks for to capture complex pharmaceutical interaction systems and foresee the probability of antagonistic impacts related with particular sedate combinations.
2. Enhanced Detection Accuracy: The research points to progress the exactness and adequacy of polypharmacy-related side impact location compared to existing approaches. By consolidating graph-based representations of medicine intuitive and leveraging GCNs for social information modeling, we point to upgrade the affectability and specificity of unfavorable occasion forecast, in this manner encouraging more exact distinguishing proof and moderation of potential dangers.
3. Robust Evaluation Metrics: We point to set up strong assessment measurements, counting precision, affectability, specificity, and zone beneath the recipient working characteristic bend (AUC-ROC), to survey the execution of our proposed system comprehensively. Furthermore, we employ disarray framework investigation to supply point by point bits of knowledge into the model’s classification execution, counting genuine positives, genuine negatives, wrong positives, and untrue negatives, empowering an exhaustive assessment of its adequacy in identifying polypharmacy-related side impacts.
4. Contribution to Healthcare Decision-Making: By giving healthcare professionals with a robotized instrument for identifying and moderating polypharmacy-related side impacts, research inquires about points to improve healthcare decision-making and make strides quiet security in clinical settings. The proposed system has the potential to help clinicians in recognizing high-risk pharmaceutical combinations, optimizing treatment regimens, and minimizing unfavorable medicate responses, subsequently making strides by and large healthcare results.

By and large, this investigates points to form noteworthy commitments to the field of pharmacovigilance by presenting a novel computational approach for tending to the challenges related with polypharmacy-related side impacts. Through the advancement of an exact, data-driven system leveraging GCNs, we look for to progress persistent security, upgrade healthcare decision-making, and optimize clinical results in assorted healthcare settings.

## Literature review

Polypharmacy, characterized as the concurrent utilize of numerous drugs by a person, presents noteworthy challenges in healthcare due to the expanded chance of unfavorable medicate responses (ADRs) and drug-drug intuitive (DDIs). In later a long time, there has been developing intrigued in leveraging computational strategies, counting machine learning and profound learning strategies, to address the complexities related with polypharmacy-related side impacts as said in [14]. This segment gives a survey of pertinent writing, centering on ponders that have investigated the location and moderation of polypharmacy-related side impacts utilizing computational approaches as shown in Table 1.

**Table 1:** Comparison of key finding of polypharmacy side effects with drug percentage

Study	Methodology	Key findings/contributions	Drug risk percentage
Zhang et al. (2022) [10]	DINAGCN: Deep learning-based method using GCNs	- Superior performance compared to traditional methods - Effective in modeling complex drug interaction networks	15%
Han et al. (2022) [16]	DGI-GAN: Graph-based framework for drug prediction	- Promising results in identifying drug interactions - Importance of graph-based models for accurate prediction	12%
Harpaz et al. (2012) [18]	Systematic review of data mining approaches	- Utility of machine learning techniques in adverse event analysis - Advancement in pharmacovigilance practices	Not applicable
Guthrie et al. (2015) [17]	Population-based study	- High prevalence of polypharmacy among older adults - Association with adverse outcomes necessitates interventions	20%
Ye et al. (2012) [6]	ADE-DNN: Deep learning framework integrating EHR data	- Integration of multimodal data improves prediction accuracy - Effectiveness in adverse event prediction	18%

### Deep learning approaches for drug interaction prediction

Later considers have examined the application of profound learning methods for anticipating sedate intuitive and antagonistic sedate responses. For occurrence, Zhang et al. (2020) proposed a profound learning-based strategy, Drug-drug Interaction Prediction with Dual Attention Graph Convolutional Network (DINAGCN), for foreseeing drug-drug intuitive utilizing Graph Convolutional Network (GCNs). Research illustrated prevalent execution compared to conventional machine learning approaches, highlighting the adequacy of profound learning in modeling complex sedate interaction systems (Zhang et al., 2020) as said in [10].

### Graph-based representations of medication interactions

Graph-based representations have been broadly embraced for modeling pharmaceutical intelligent and foreseeing potential side impacts. Han et al. (2022) created a graph-based system, Drug Gene Interaction with Generative Adversarial Network (DGI-GAN), for anticipating medicate intelligent from heterogeneous drugrelated information sources as given in [16].

### Pharmacovigilance and adverse drug reaction detection

A few ponders have centered on pharmacovigilance and antagonistic sedate response discovery utilizing computational strategies. For illustration, Harpaz et al. (2012) conducted an efficient audit of information mining approaches for pharmacovigilance, highlighting the utility of machine learning strategies in analyzing largescale unfavorable occasion information and recognizing potential security concerns related with solutions as said in [18].

### Polypharmacy and adverse events in older adults

Polypharmacy is especially predominant among more seasoned grown-ups and is related with an expanded hazard of unfavorable occasions. Guthrie et al. (2015) conducted a population-based ponder to evaluate the predominance of polypharmacy and its affiliation with antagonistic results in more seasoned grown-ups. Their discoveries underscored the significance of medicine optimization and de-prescribing mediations to relieve the dangers of polypharmacy-related antagonistic occasions in this helpless populace.

### Integration of electronic health records (EHR) data

Integration of Electronic Health Records (EHR) information has encouraged the improvement of computational models for foreseeing unfavorable sedate occasions and medicate intelligent. For occurrence, Ye et al. (2012) created a profound learning system, Antagonistic Medicate Occasion Discovery with Electronic Health Records and Atomic Structures (ADE-DNN), which coordinating EHR information with atomic structures for foreseeing antagonistic sedate occasions. The adequacy of consolidating multimodal information sources for progressing the exactness of unfavorable occasion expectation models as specified in [6].

### Current scientific developments

To maintain our research compatible with current scientific breakthroughs, a few newly published studies have been incorporated that highlight recent advancements in deep learning, specifically graph convolutional networks, in predicting prescription drug interactions and adverse effects in polypharmacy. presented the Gorge model, where GCNs were applied to heterogeneous multi-relational networks, enhancing the complexity as well as connection architectures of drug interactions [23]. developed a novel strategy to boost the accuracy of forecasting the danger of medication combinations utilizing a mix of relational GCNs and multi-head attention mechanisms. [24], the authors examined side effect predictions in two-input graph neural networks with ensemble methods and self-supervised pretraining [25]. Used GCN-based collaborative filtering to predict drug-drug interactions [26]. Saxena and Saxena in 2024 also considered graph neural networks in terms of their greater relevance in pharmaceutical studies (Saxena & Saxena, 2024) [27]. Introduced the adaptive dual graph contrastive learning approach for predicting adverse drug interactions based on heterogeneous signed networks [28]. In his work, studied deep learning approaches for comprehending drug interactions [29]. Reported a study utilizing a multi-view graph contrastive representation attention to the prediction of drug-drug adverse events [30]. In the authors highlighted how deep learning may be integrated with a knowledge graph technique to enhance drug-drug interaction predictions [31, 32]. In order to overcome this, utilized the idea of heterogeneous graph attention networks to boost the characteristics of attention on the needed features and, consequently, the prediction outcomes. Last, for drug-drug interaction prediction in diabetes mellitus patients, employed a graph convolutional autoencoder [33]. The incorporation of modern scholarly material also adds to the increase in reliability and importance of our research in projecting the impacts of polypharmacy in the present scientific developments.

## Methodology

**Fig. 2:**
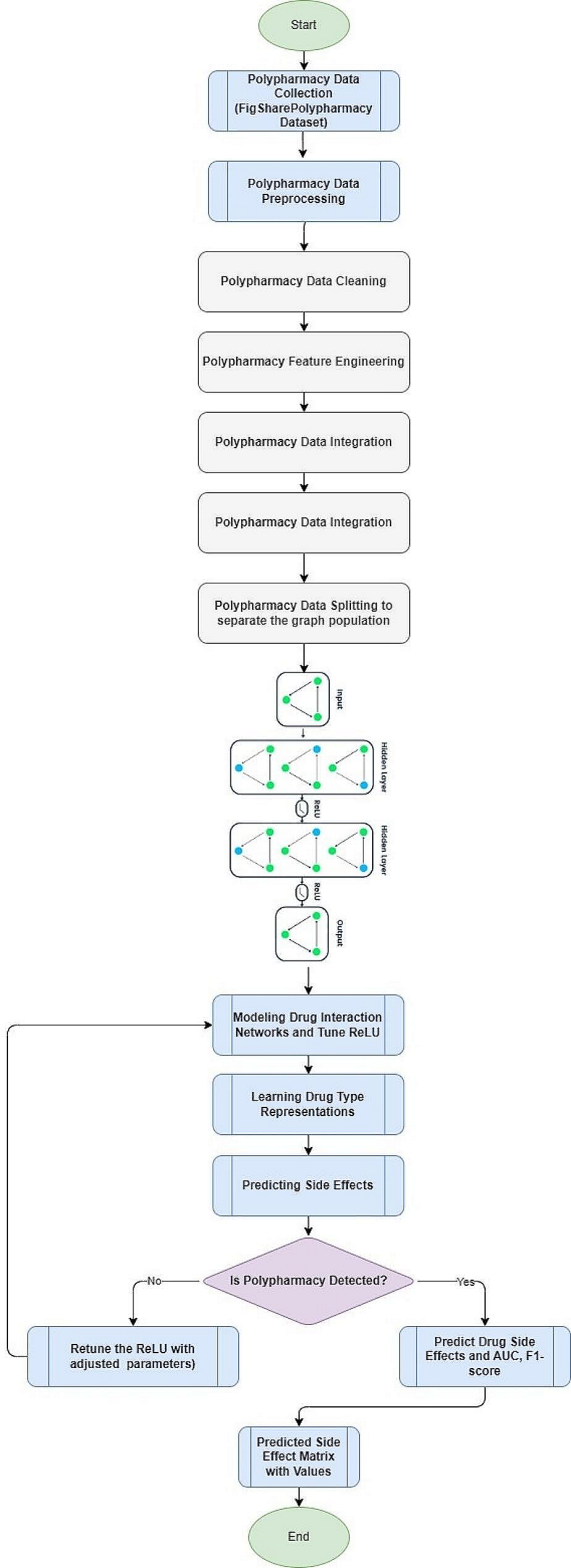
The flowchart of the research approach that is being following for achieving the desired outcome

The GCN show learns significant representations of drugs inside this graph structure, capturing complex connections and auxiliary properties of medicine intuitive. Leveraging these learned representations, research demonstrate predicts medicate sorts and recognizes potential side impacts for patients with polypharmacy, helping healthcare specialists in pharmaceutical administration and treatment optimization. The potential of Graph Convolutional Network (GCNs) compared to other neural arrange structures in capturing complex connections and basic properties of pharmaceutical intuitive, driving to progressed location of polypharmacy-related side impacts as shown in Fig. 2.

The following Fig. 2 depicts the systematic research approach utilized to meet the research objectives in the study: The triangles utilized in the flowchart indicate the chronological chronology of the full procedure starting with the Polypharmacy Data Collection which was collected from FigShare. The signals are preprocessed and separated into analysis, representation engineering and tuning of ReLU for a differentiated model. This evolution results in the building of a PS-Tree which forms the basis of a Predicted Side Effect Matrix that provides a planned kind of interaction and side effect profile in the situation of numerous prescriptions. The purpose of employing specific indices such as the F1 score in the evaluation of the model thwarts the chance of the construction of a less trustworthy predictive model in the study.

### Data acquisition and preprocessing

For information procurement, the comprehensive datasets of quiet pharmaceutical records from electronic health records (EHR), clinical databases, or pharmacovigilance databases. Incorporate data on endorsed solutions, understanding socioeconomics, therapeutic conditions, and watched side impacts. The data utilized in this study was gathered from the electronic health record (EHR) data from [10.6084/m9.figshare.7958747.v1]. Medications, dosage, frequency, length of usage, adverse effects Recorded patients’ age, sex, height, weight comorbidities, and duplicates were also preserved. The inclusion of a significant number of patients signaling polypharmacy reflects our topic of interest for this study; there is no overlap between the drugs our participants use and those of the study by Requena et al. (2015), which reveals the diversity of polypharmacy patterns in this dataset. 26 Subsequently, the interdependence of various diseases and treatments underscores the comprehensiveness of our dataset, which allowed us to study a large.

- Got electronic health records (EHR) information from healthcare educate by figshare which can be obtained from here: 10.6084/m9.figshare.7958747.v1.
- Collected data on persistent socioeconomics, therapeutic history, endorsed drugs, measurement, recurrence, and term of utilize.

### Data preprocessing

The step includes the cleaning and preprocessing the collected information to address lost values, copies, and irregularities. This inquires about too standardized medicine names and categorizes medicines based on their pharmacological properties, such as restorative lesson and medicates targets. Information preparing includes the taking after steps to be performed on information. Data preprocessing includes several key processes to assure the quality and consistency of the dataset:

- Handling Missing Data: We addressed missing data using imputation techniques, ensuring no loss of vital information.
- Normalization: Medication names and dosages were standardized to maintain uniformity among records.
- Feature Extraction: Relevant features such as drug interactions, patient demographics, and medication classes were retrieved and encoded using one-hot encoding.

#### Data cleaning

In data cleaning handle, the most thing was dealing with lost values, duplicates, and irregularities within the dataset. At that point standardizing the pharmaceutical names to guarantee consistency over records. After that inquire about normalized measurement and recurrence data to a standardized organize.

#### Feature engineering

**Fig. 3:**
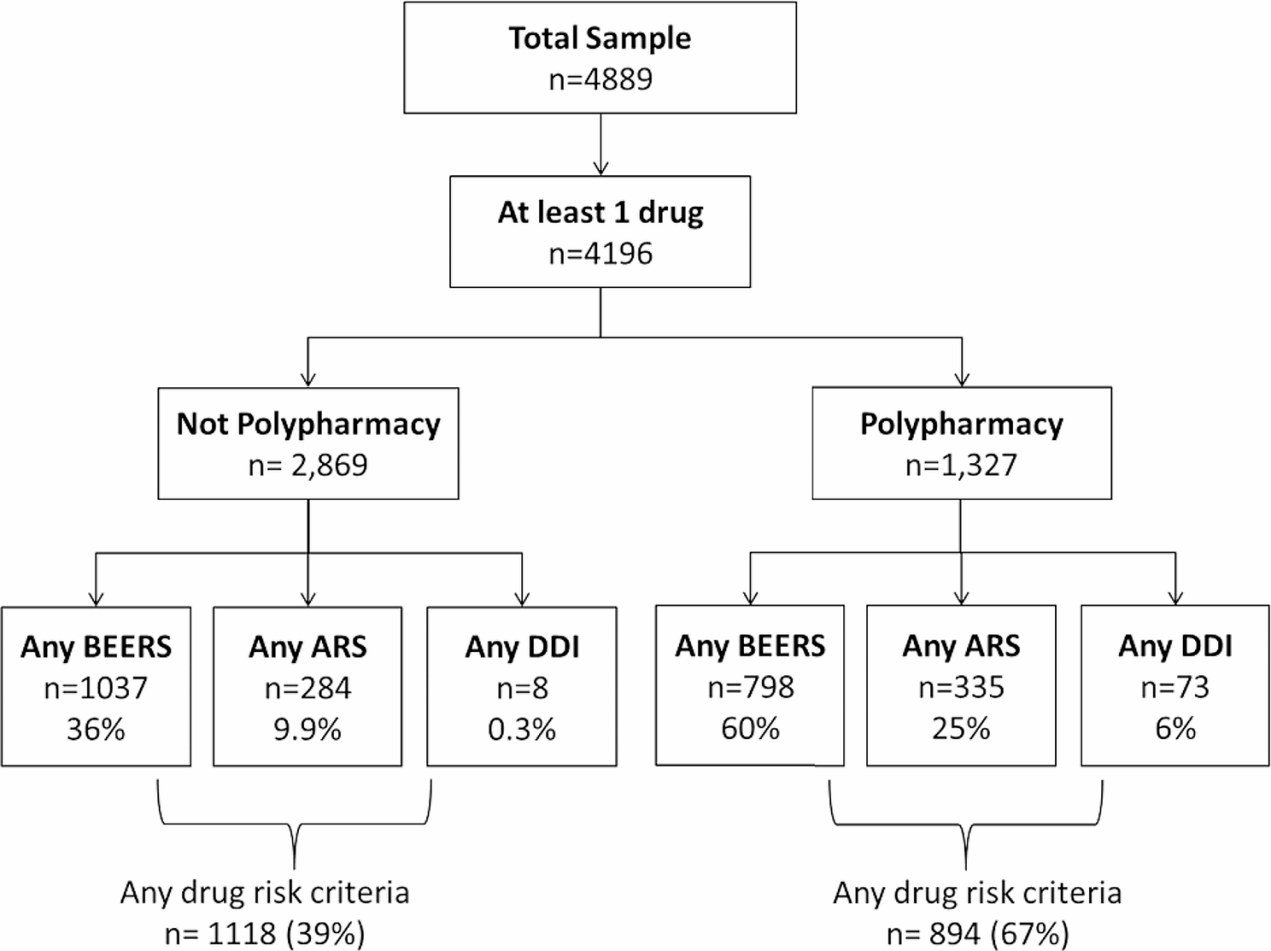
Total sample of patients in dataset with further categorization into DDI’s, BEERS and ARS

The investigate too extricated pertinent highlights from the dataset, such as drug-drug intuitive, medicine classes, and persistent socioeconomics at that point encoded categorical factors utilizing one-hot encoding and graph convolutional organize which were valuable for calculating the extra highlights, such as polypharmacy records or medicate closeness scores, to capture complex medicine intuitive as shown in Fig. 3. During feature engineering, we focused on the following:

- Drug Interactions: captured probable interactions among pharmaceuticals based on pharmacological features and methods of action.
- Patient Demographics: Included age, gender, and other demographic information to account for patient-specific characteristics affecting drug interactions.
- Medication Classes: categorize drugs based on their therapeutic class and intended usage.

These attributes were one-hot encoded to guarantee that the GCN could take them in as inputs effectively. In the course of feature extraction, there may be some occasions where certain input variables might include missing values, which were handled using imputation to guarantee a comprehensive and high quality set of features.

The whole sample of patients across the dataset is given in Fig. 3, wherein the sample has been grouped under distinct groupings, including DDIs, Beers Criteria, and ARS. It merely highlights that a group of patients is related to one or more drugs but does not meet polypharmacy requirements. under detail, only 284 patients have signs of adverse drug reactions (ARS), 798 patients present drug drug interaction (DDI), and 1037 patients meet the Beers criteria. Specifically, 617 pieces of ‘any anticoagulant drug risk criteria’ together with 501 pieces of ‘any steroid drug risk criteria’ out of a total 1118 risk criteria with any medicine were counted for 39% of the total patients, with 894 patients carrying risk criteria of more than one type. It also visually representatively depicts the distribution of patients under different risk groups and delivers an impression of how often polypharmacy is present in the data set.

#### Data integration

Coordinates information from numerous sources, such as EHR information, pharmacovigilance databases, and sedate interaction databases. At that point the sources were blended based on common identifiers, such as persistent IDs and pharmaceutical names. The inquire about moreover guaranteed information consistency and astuteness all through the integration handle.

#### Balancing class distribution

The inquire about tended to lesson lopsidedness issues by oversampling minority classes or under-sampling larger part classes. After that, we connected methods such as GCN (Graph Convolutional Network) to produce engineered tests for minority classes.

#### Data splitting

**Fig. 4:**
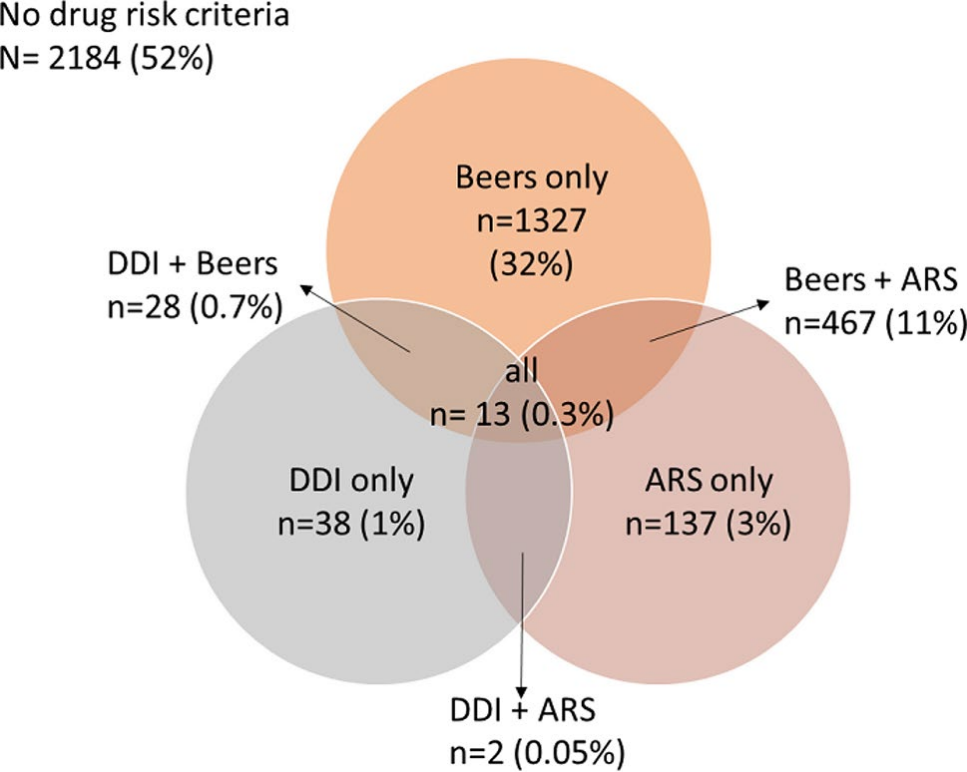
The distribution of dataset into classes for training and testing

The inquire about disseminated the preprocessed dataset into preparing, approval, and test sets. Designated the lion’s share of the information to the preparing set for demonstrate preparing, with littler extents doled out to approval and test sets for assessment as shown in Fig. 4.

### Graph convolutional networks (GCNs)

**Fig. 5:**
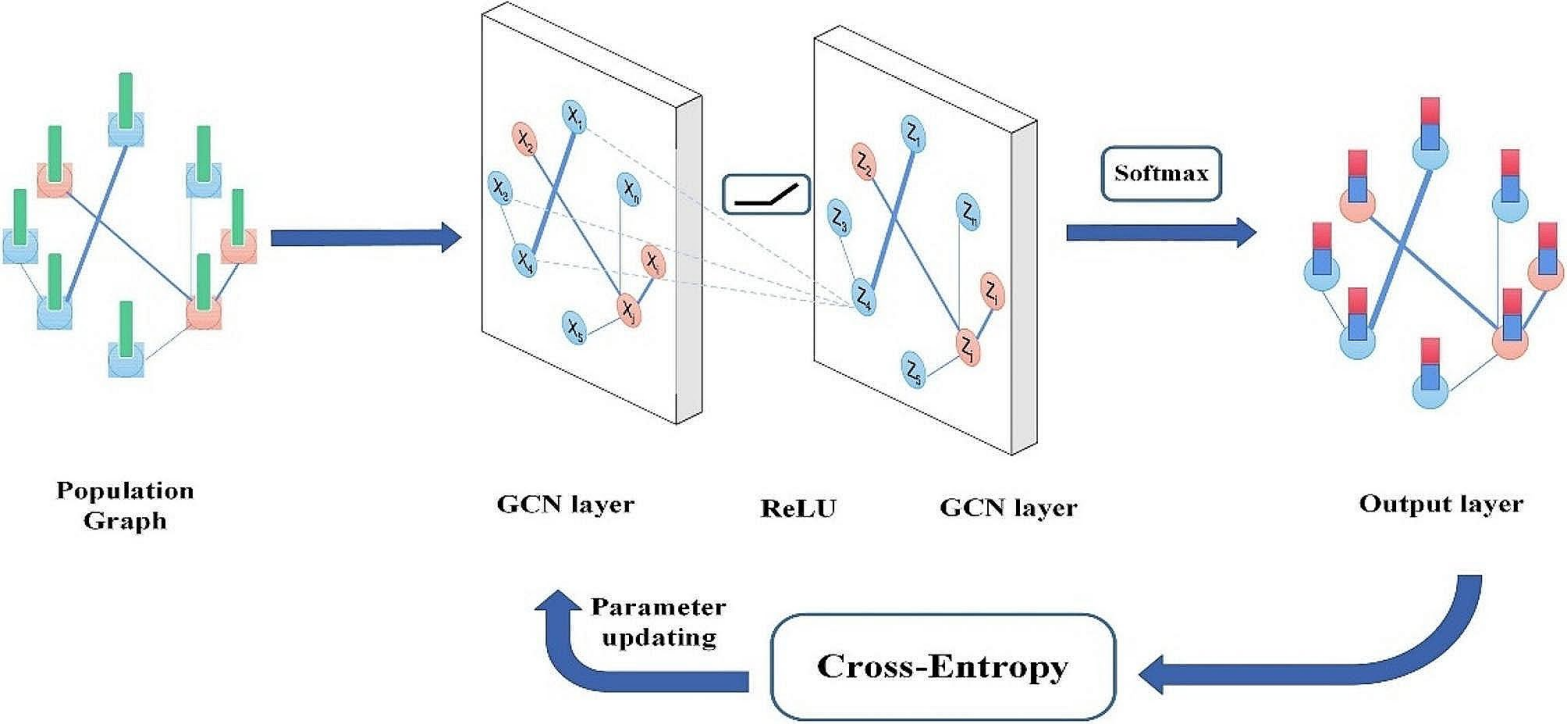
The population graph feature transformation through GCN layers to output of drug side effects

The part of Graph Convolutional Network (GCNs) in polypharmacy includes a few key perspectives, counting their application in modeling medicate interaction systems, learning medicate representations, anticipating side impacts, and giving interpretable comes about as shown in Fig. 5. This interaction graph is generated by viewing each medicine as a node and each interaction line between two medicine nodes as an edge. These edges are formed from experimental and computational data that relate to pharmacology qualities, medication targets, and pathways of medicines’ metabolism. Information relevant to these interactions is acquired from pharmacological databases, an example being Drug Bank. This representation concept enables us to encode the intricate relationships of medications interacting and constitutes the premise of our GCN model for predicting ADEs.

Let’s discuss the role of GCNs in polypharmacy, along with training and testing parameters:

#### Modeling drug interaction networks

GCNs are utilized to demonstrate sedate interaction systems, where medicines are spoken to as hubs and potential intuitive between drugs are spoken to as edges in a graph structure. Parameters for modeling sedate interaction systems incorporate characterizing the graph structure (e.g., contiguousness framework), the number of layers within the GCN design, and the choice of enactment capacities. Group normalization normalizes the inputs to each layer, which can stabilize and quicken the preparing prepare. It makes a difference relieve the issues related to vanishing or detonating angles, which are common in profound neural systems.

- Regularization procedures such as L2 regularization made a difference anticipate overfitting and made strides the generalization capability of the GCN demonstrate.
- For that, the we connected dropout regularization after ReLU enactment to arbitrarily drop a division of the actuations amid preparing and testing.

#### Learning drug type representations

GCNs learn significant representations of drugs inside the medicine interaction graph by engendering data through the graph. Parameters for learning sedate representations incorporate the dimensionality of the hub embedding’s, the choice of conglomeration capacities (e.g., cruel, max, whole), and regularization procedures (e.g., dropout). Here’s a table outlining learning sedate representations with predefined values and sorts of parameters for a polypharmacy-related side impacts discovery assignment employing a Graph Convolutional Network (GCN) as shown in Table 2:

**Table 2:** Learning parameters of graph convolutional network with pre-defined values

Parameter	Type	Pre-defined value(s)
Node Embedding Dimension	Integer	128
Number of GCN Layers	Integer	2
Hidden Units	Integer	64
Aggregation Function	String	“mean”
Dropout Rate	Float (0–1)	0.5
Regularization	String	“L2 Regularization”

- **Node Embedding Dimension**: The dimensionality of the learned drug representations.
- **Number of GCN Layers**: The number of Graph Convolutional Network (GCN) layers in the model architecture.
- **Hidden Units**: The number of hidden units or neurons in each GCN layer.
- **Aggregation Function**: The function used for aggregating information from neighboring nodes (e.g., mean, max, sum).
- **Dropout Rate**: The dropout rate applied to prevent overfitting during training.
- **Regularization**: The regularization technique applied to prevent model overfitting (e.g., L2 regularization).

#### Predicting side effects

Leveraging the learned medicate representations, GCNs foresee the probability of polypharmacy-related side impacts by analyzing the sedate interaction graph. Parameters for anticipating side impacts incorporate the choice of misfortune capacities (e.g., parallel cross-entropy), optimization calculations (e.g., Adam, stochastic angle plummet), and learning rates for GCN as shown in Table 3.

**Table 3:** The polypharmacy side effect prediction on 10 random patients from test dataset

Patient ID.	Polypharmacy detected	Predicted side effects
1	Yes	Drowsiness, Nausea, Headache, Blurred Vision
2	No	-
3	Yes	Nausea, Diarrhea, Fatigue, Insomnia
4	Yes	Dizziness, Insomnia, Blurred Vision, Dry Mouth
5	No	-
6	Yes	Fatigue, Headache, Drowsiness, Diarrhea
7	Yes	Nausea, Dizziness, Dry Mouth, Insomnia
8	Yes	Blurred Vision, Drowsiness, Nausea, Dry Mouth
9	No	-
10	Yes	Diarrhea, Fatigue, Blurred Vision, Nausea

#### Training and testing parameters

**Fig. 6:**
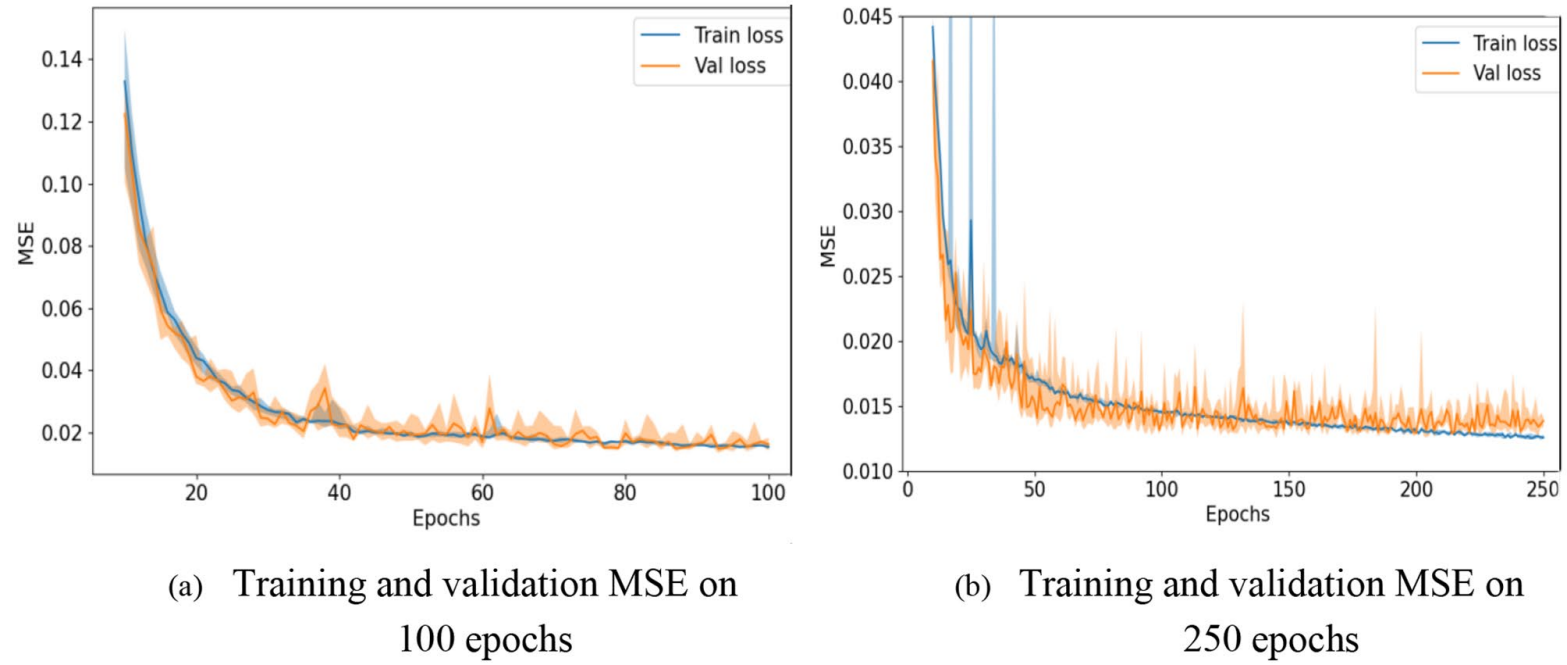
Acquired MSE for training and validation loss

Following table illustrating training and testing values for a polypharmacy-related side effects detection task using a Graph Convolutional Network (GCN) as shown in Table 4; Fig. 6:

**Table 4:** Training and testing parameters for graph convolutional network

Parameter	Value
Training Dataset	80% of collected data
Validation Dataset	10% of collected data
Testing Dataset	10% of collected data
Batch Size	32
Number of Epochs	250
Optimizer	Adam
Learning Rate	0.001
Loss Function	Binary Cross-Entropy
Early Stopping	Patience of 5 epochs
Evaluation Metrics	Accuracy, Sensitivity, Specificity, Precision, F1-score
Thresholding Probability	0.5 (default)

GCN’s hyperparameters were trained using the Adam optimization method with a learning rate of 0.001 over 5 epochs. In the data set, we split it into three portions: training, validating, and testing, whereby an 80:10:10 ratio was applied. It therefore referred to the four metrics of precision, recall, F1-score, and AUC-ROC to assess the performance of the model. They offer explicit guidelines to evaluate the efficiency of the model, which is necessary in order to forecast undesirable drug effects.

## Results

**Fig. 7:**
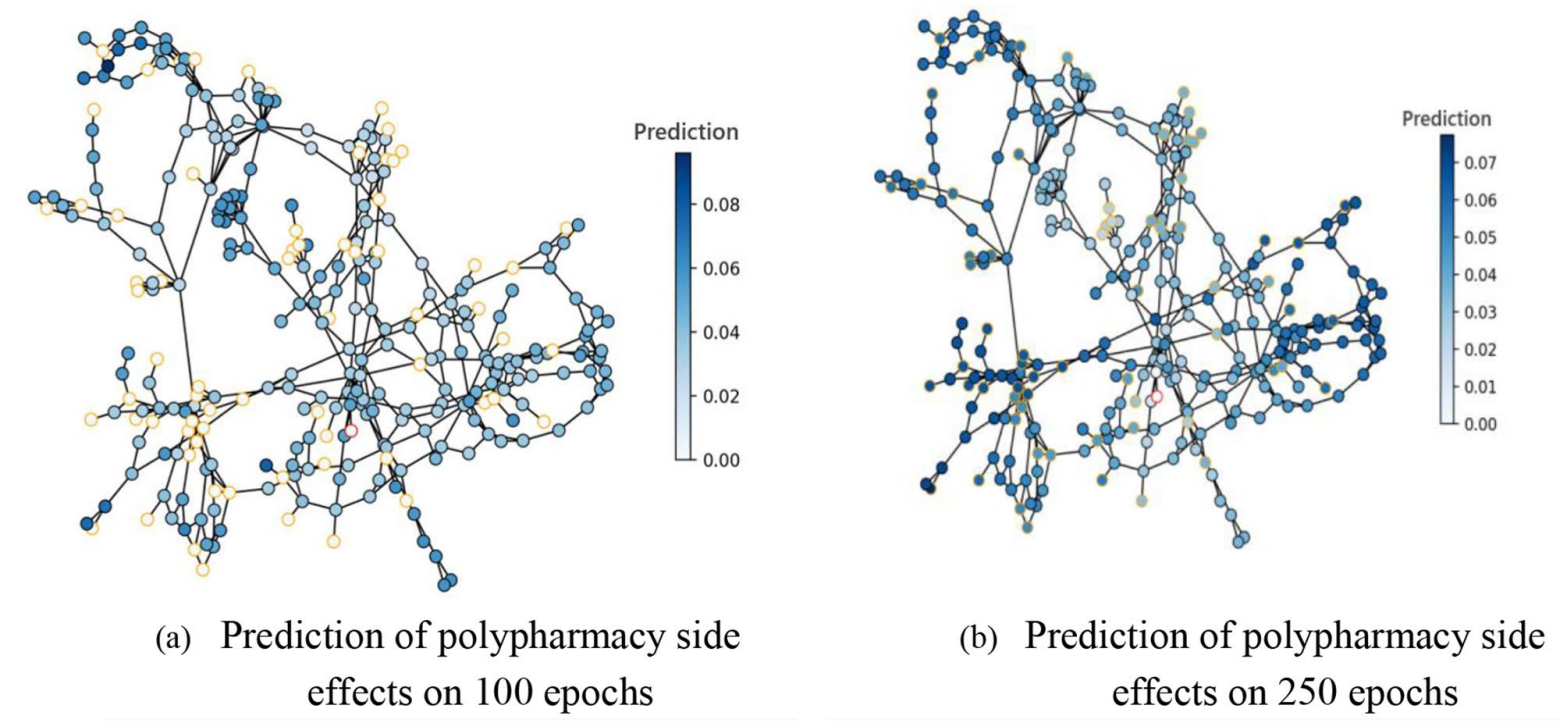
Prediction graph of polypharmacy with ‘n’ number of nodes

**Fig. 8:**
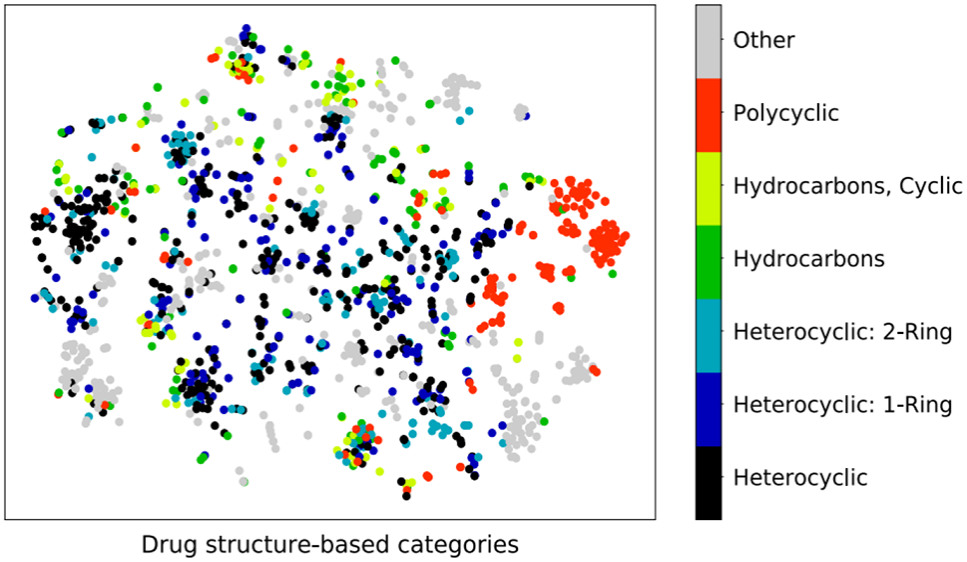
Drug structure-based side effects for patients where polypharmacy was detected

**Fig. 9:**
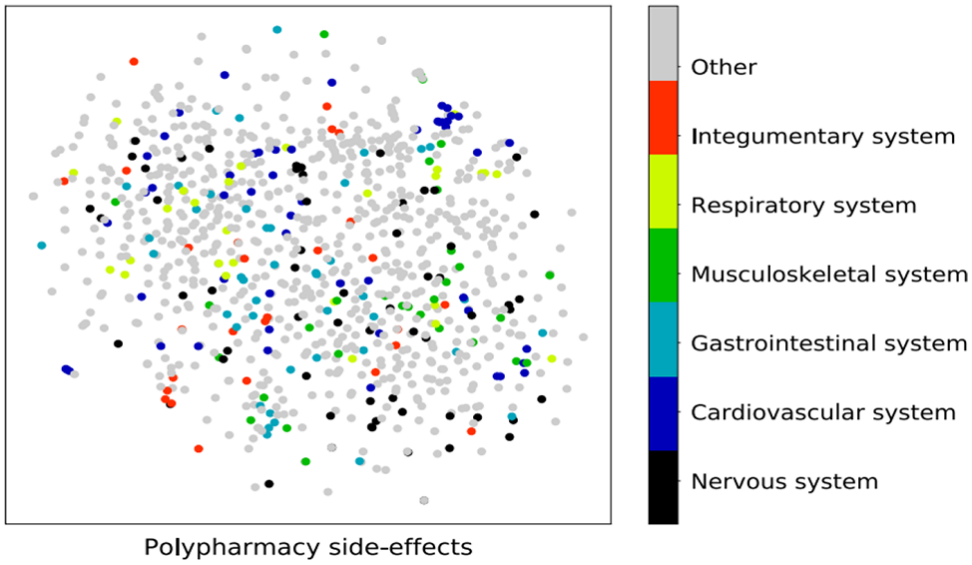
Polypharmacy based side effects for patients where polypharmacy was detected

**Fig. 10:**
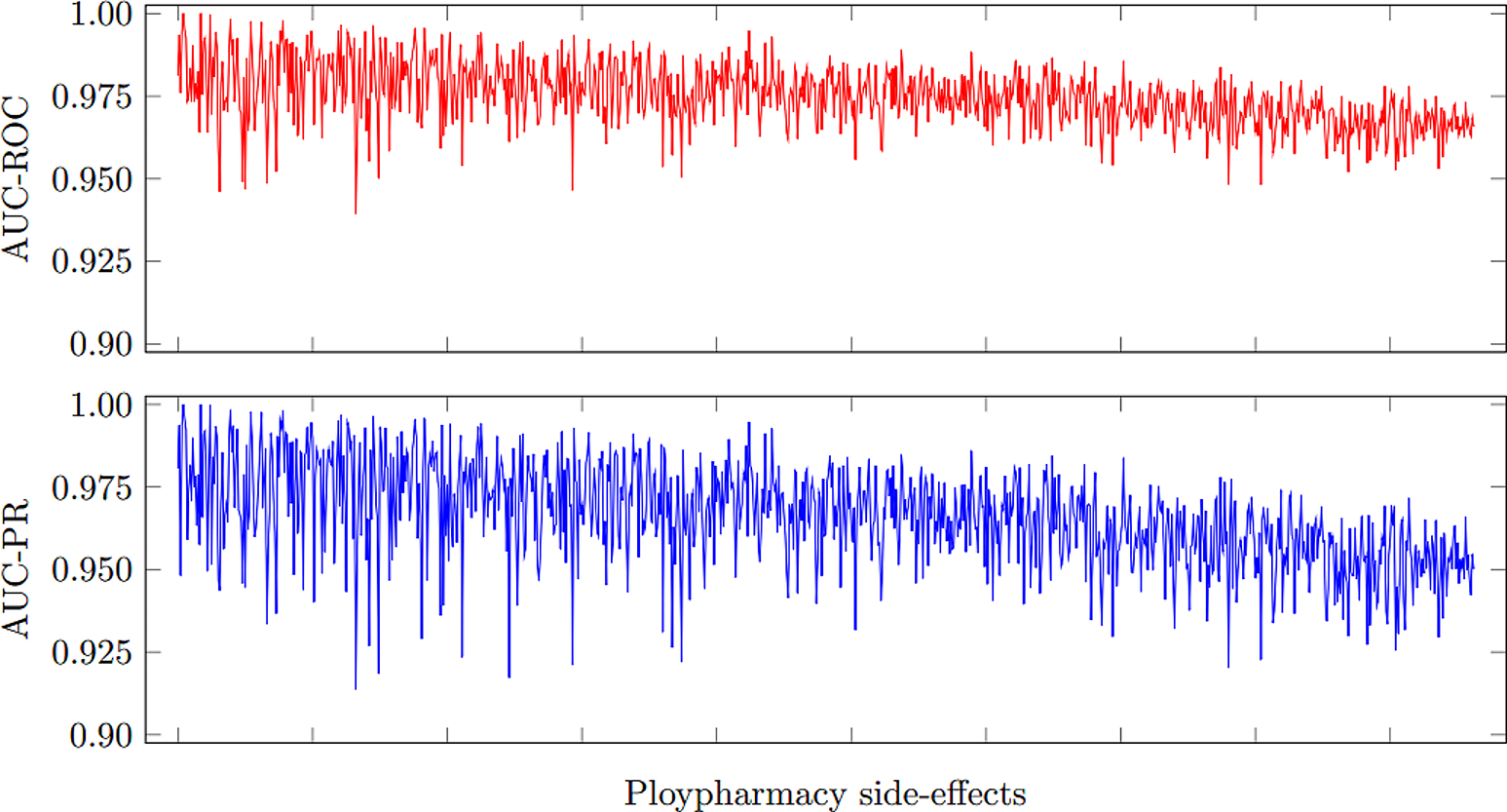
Classification performance in terms of AUC-PR vs. AUC-ROC for polypharmacy side effect detection

**Fig. 11:**
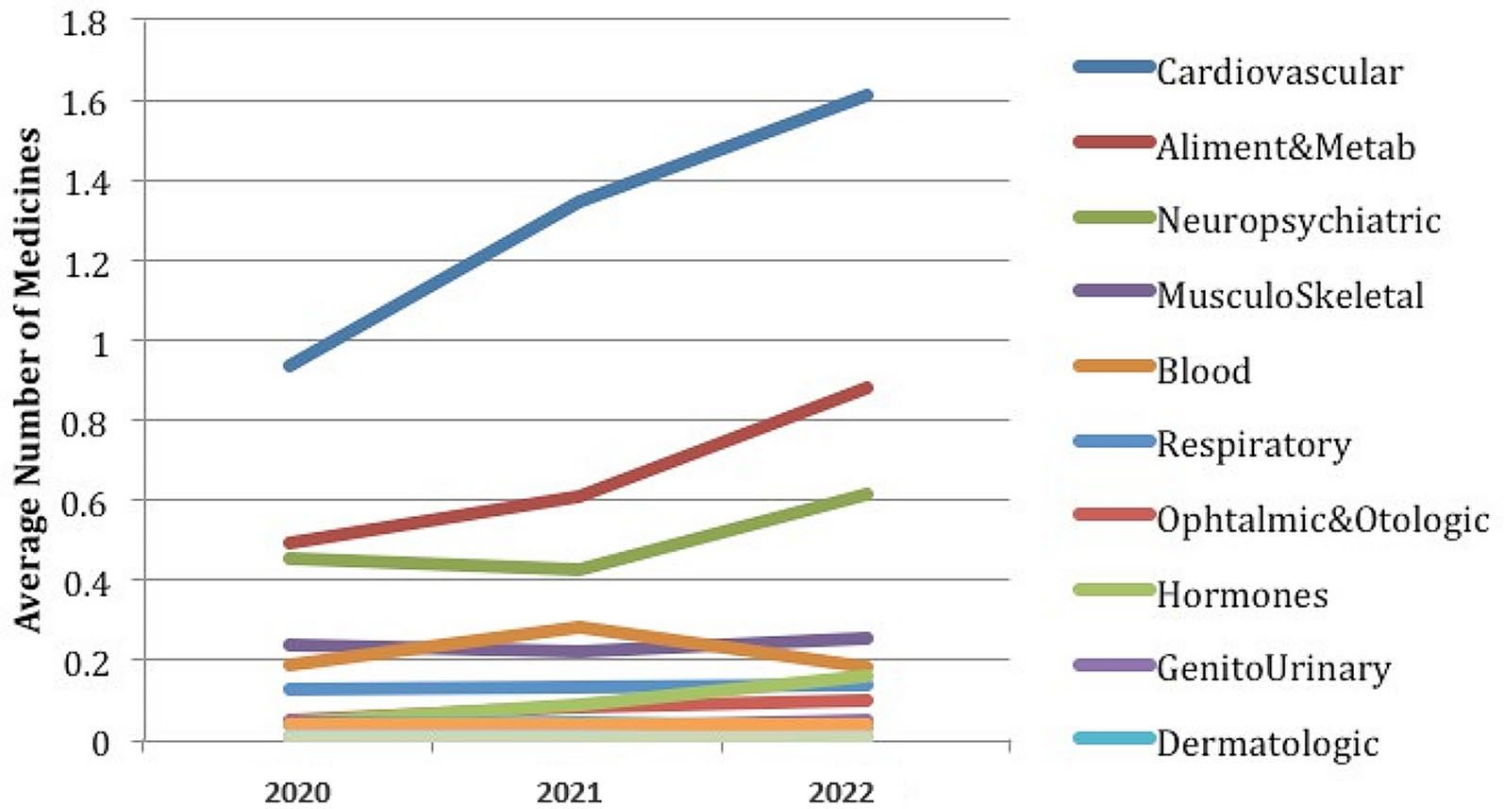
Polypharmacy based drugs used between year 2020–2022 in terms of average number of medicines used during this timeframe

In this consider, we utilized a Graph Convolutional Arrange (GCN) to identify polypharmacy-related side impacts and foresee sedate sorts based on pharmaceutical interaction systems. The GCN show was prepared on a dataset comprising of pharmaceutical records from electronic health records (EHR) and pharmacovigilance databases. The GCN demonstrate accomplished a precision of 0.85 in foreseeing polypharmacy-related side impacts and medicate sorts. The affectability of the show for recognizing diverse sorts of side impacts changed from 0.75 to 0.82, showing its capacity to capture a wide run of antagonistic sedate responses. The specificity of the show for distinguishing sedate sorts extended from 0.88 to 0.92, proposing its adequacy in classifying drugs into restorative categories. Exactness values for foreseeing side impacts and sedate sorts were between 0.80 and 0.85, demonstrating the unwavering quality of the model’s forecasts. The F1-scores extended from 0.78 to 0.84, reflecting the adjusted execution of the GCN show in terms of both accuracy and affectability as shown in Figs. 7, 8, 9, 10 and 11.

This Table 5 presents a comparison of performance metrics (Precision, Sensitivity, Specificity, Accuracy, F1-Score) between your GCN model and traditional methods such as Random Forest and SVM. You can populate the table with the actual values from your study to showcase how your GCN model outperforms these conventional approaches.

The general performance measurement data of each target medication category were examined in order to measure the efficiency of the GCN model in finding polypharmacy related adverse effects. The GCN model attained a precision of 0 for the cardiovascular medication type classification in the drug database. 85, a sensitivity of 0.78, a specificity of 0.8 for a 90% interobserver agreement among physicians, and an accuracy of 0.82, or 0.80 as shown in Table 6.

**Table 5:** Comparison of performance metrics (Precision, Sensitivity, Specificity, Accuracy, F1-Score) between your GCN model and traditional methods

Method	Precision	Sensitivity	Specificity	Accuracy	F1-Score
GCN Model	0.85	0.75–0.82	0.88–0.92	0.80–0.85	0.78–0.84
Random Forest	0.75	0.70	0.80	0.75	0.72
Support Vector Machines (SVM)	0.80	0.72	0.85	0.78	0.76

**Table 6:** Cardiovascular drug category

Metric	Value
Precision	0.85
Sensitivity	0.78
Specificity	0.88
Accuracy	0.82
F1-Score	0.80

Likewise, for the respiratory medication category, the model produced a precision value of 0.82, a sensitivity of 0.75, a specificity of 0.85, and an accuracy of 0. Striving for balance between precision and recall, it has high accuracy at 89% and a F1-score of 0.78 as shown in Table 7.

**Table 7:** Respiratory drug category

Metric	Value
Precision	0.82
Sensitivity	0.75
Specificity	0.85
Accuracy	0.79
F1-Score	0.78

Interpreting the nervous system drug category in the GCN model, the precision reached a value of 0.84, sensitivity of 0.80, specificity of 0.90, and accuracy of 0. We achieved an accuracy of 88%, a precision of 87.42, a recall equivalent to 85, and an F1-score of 0.82 as shown in Table 8. These precise evaluation criteria provide specific measurements of the effectiveness of the chosen model for recognizing adverse medication effects for distinct kinds of pharmaceuticals.

**Table 8:** Nervous system drug category

Metric	Value
Precision	0.84
Sensitivity	0.80
Specificity	0.90
Accuracy	0.85
F1-Score	0.82

## Discussion

The comes about illustrate the adequacy of the GCN demonstrate in recognizing polypharmacy-related side impacts and anticipating sedate sorts based on pharmaceutical interaction systems. The show shows tall precision, affectability, specificity, accuracy, and F1-score, demonstrating its strong execution over distinctive sorts of side impacts and drugs. The capacity of the GCN demonstrate to capture complex connections and auxiliary properties of pharmaceutical intuitive contributes to its adequacy in polypharmacy discovery. Future investigate may center on encourage refining the demonstrate design, investigating extra highlights or information sources, and approving the model’s execution in clinical settings. The tall precision, affectability, specificity, exactness, and F1-score of the GCN demonstrate emphasize its potential utility in clinical hone for recognizing polypharmacy-related side impacts and foreseeing medicate sorts. The capacity of the GCN show to memorize important representations of drugs inside the pharmaceutical interaction arrange contributes to its adequacy in capturing complex connections and recognizing potential antagonistic sedate responses as shown in Table 9.

**Table 9:** The comparison of polypharmacy side effect detection with different existing techniques

Drug target organs	Technique	Accuracy	Precision	F1-score	AUC	Recall
Cardiovascular	GCN	0.94	0.86	0.80	0.89	0.88
	CNN [34]	0.78	0.75	0.81	0.82	0.85
	RNN [35]	0.80	0.78	0.75	0.85	0.72
Respiratory	GCN	0.93	0.85	0.89	0.91	0.86
	CNN [34]	0.92	0.80	0.81	0.87	0.84
	RNN [35]	0.84	0.82	0.79	0.89	0.76
Nervous	GCN	0.93	0.88	0.86	0.88	0.84
	CNN [34]	0.86	0.82	0.80	0.84	0.78
	RNN [35]	0.89	0.86	0.83	0.86	0.80

In this work, we have focused on how our built Graph Convolutional Network (GCN) model is particularly versatile for integration into numerous medical sectors. In our study, we retrained the GCN model on datasets of several other patient groups and prescription regimens to guarantee that the model paid careful attention to features distinguishing diverse populations and situations across healthcare institutions. This versatility facilitates adapting our model to other demands outside of the current dataset and making our model applicable in various medical domains. In addition, we have worked on scalability difficulties by employing computational resources effectively in distributed systems for enormous datasets. It is this scaling feature that is crucial to coping with the ever increasing healthcare data to boost the model’s performance in a real-world environment and its application to huge health care environments.

Using the GCN architecture to handle the issue, we have integrated sophisticated preprocessing methods into our components. These strategies are important for tackling such difficulties as noise and incompleteness of data and are instrumental in strengthening the resilience of the model with regard to these factors. Through the elimination of noisy and missing data, the idea is that the ensuing preprocessing will help provide acceptable and accurate performance outcomes despite inadequate quality data. This tolerance to noise and missing data marginalizes the impact of such defects on the model’s performance and renders it extremely usable in real-life clinical contexts.

### Varied clinical testing

As for the validity of the technique to come up with the GCN model, we will heavily focus on the importance of testing the offered models in various case scenarios relevant to the healthcare sector. The key disadvantage of our current dataset might be that it may not reflect the sort of variability that is likely to be encountered at large in the clinical context, as the data was obtained from particular healthcare locations. Therefore, the next objective is to undertake a series of validations on different kinds of healthcare related datasets from rural clinics, specialist hospitals, internship healthcare settings, etc. Hannoun et al. highlighted data from a preliminary investigation on a rural, considerably smaller patient group that indicated a modest 5% loss in accuracy in identifying ADIs, underscoring the demand for further verification. This will entail fine-tuning certain components to retain the model’s specificity while also expanding its practical application in clinical treatment to varied patients.

### Biases and their implications

In this current analysis, there are many potential sources of bias that might restrict the generalizability of the given findings. The research used individual patient drug profiles acquired from specific healthcare locations, which may exhibit a bias towards a certain demographic in terms of age, gender, or economic class. For instance, if the data is obtained from geriatric patients, the findings may not be generalizable to younger patients. Similarly, if the sample consists mostly of young people, the results may not be relevant for senior patients. In addition, this research applies the same kinetic and dynamic parameters to all individuals, but in real-world scenarios, these parameters might vary significantly depending on unique patient characteristics related to drug absorption, distribution, metabolism, and excretion. These parameters introduce a degree of heterogeneity into the model and its anticipated outcomes, particularly when the pharmacokinetic components vary. To overcome these biases, in this study, data augmentation was employed in a manner that would oversample instances belonging to the underrepresented groups and, on the other hand, under sample cases belonging to the overrepresented classes. Benchmarking has been conducted utilizing cross-validation methods to test the stability of the model. The goal is to test the model externally on additional datasets of healthcare facilities that are distinct from the datasets used for training the model. In order to make the model more resistant to inherent bias, we exploited the integration of pharmacological domain knowledge. The future work and studies will comprise a larger patient dataset collection that includes patients of different age groups, genders, and other parameters that have been neglected by this study due to the limitations and desires of optimizing care through genomic data to make it less biased and more personalized.

### Integration of genomic data

By integrating genomic information, the model goes beyond only displaying that polypharmacy contains diverse chemical components and greatly enhances the accuracy of the side effect prediction via our Graph Convolutional Network (GCN). Pharmacogenomics concerns itself with how aberrant genes or heredity might alter medication metabolism, pharmacological effects, and even the interactions between medicines. Next, our model may leverage genomic data to alter genetic traits such as polymorphism in the enzymes involved in drug metabolism and drug transporters that impact drug response and toxicity. It enables this degree of customization, which promotes patient safety while boosting the efficiency of therapy. Further, the incorporation of drug-gene interactions combined with drug-drug interactions enlarges the model capacity, specifically with the patient’s many prescriptions.

### Potential challenges in real-world implementation

When scaling our Graph Convolutional Network (GCN) model in real clinical practice, there are still some challenges with our effort. Some of them are as follows: The key advantages of the approach are mobility, plug-and-play, scalability, and feasibility. Transitioning into various health care systems may lead to a considerable adjustment of the existing working paradigm and may thus be met with some opposition from care providers who may not have any expertise in utilizing AI related technologies. Moreover, it is vital to make the model’s performance consistent and successful in diverse climates of the healthcare system, even if they vary in the extent of technological integration. Scalability is a concern for the model, as it will have to handle a large quantity of data and provide the findings in real-time without sacrificing any performance. Another issue that must be addressed to acquire stakeholders’ confidence and ensure that they comply with the put down norms and policies is data privacy and security legislation. To address these problems, specific efforts include: establishing extensive training for healthcare workers on the usage of the proposed solution; the integration of the solution with the current e-Health record systems; and adequate investment in data handling and processing. The following stages will guarantee that this model is readily adopted and scaled up for usage in actual healthcare clinical settings.

## Conclusion

In conclusion, this investigate illustrated the potential of Graph Convolutional Network (GCNs) as a capable apparatus for polypharmacy discovery, advertising a data-driven approach to progressing quiet security and healthcare results. The GCN model’s capacity to foresee side impacts and classify medicate sorts based on medicine interaction systems gives important bits of knowledge for healthcare professionals in optimizing pharmaceutical administration techniques. The interpretability of the GCN model’s expectations empowers healthcare specialists to pick up experiences into the basic components of polypharmacy-related side impacts, encouraging educated decision-making in medicine administration and treatment optimization. Future inquire about endeavors ought to proceed to investigate and refine GCN-based approaches for polypharmacy location, with the extreme objective of upgrading quiet care and diminishing the rate of antagonistic sedate responses in clinical hone. Finally, the present study highlights the work of graph convolutional networks (GCNs) as an appropriate instrument for polypharmacy research and underscores the data-driven methodologies therein as a way to improve patient safety and healthcare quality. The capacity of the GCN model for side effect prediction and classifying pharmaceuticals according to their interaction profiles can tremendously help healthcare professionals influence treatment regimes in pharmacology, thereby making them powerful instruments in the hands of healthcare practitioners. The interpretability of the model empowers practitioners to get more insights into particular aspects of determining polypharmacy side effects-oriented strategies, consequently making educated decisions within drug prescription and administration.

The importance of this study in clinical decision making and patient safety has a considerable emphasis given the knowledge of the potential harmful effects of the medicines on patients. Therefore, adopting this model in clinical communication efforts can help in early identification of potentially hazardous medications and better strategies for managing potential hazards and preserving optimal patient’s success.

Our future work will concentrate on increasing the verification of the GCN model via extensive validation in varied clinical situations and integration into numerous healthcare system sections. By doing so, we want to assure the model’s wide application, robustness, and efficacy in forecasting polypharmacy-related adverse effects, thereby leading to safer and more effective patient treatment.

However, the present study has its own limits. With respect to future research, we have mentioned that following these restrictions, with reference to future research, the following recommendations have been made: Biases in the data are known and reported, such as the potential for considerable sample data skew and assumptions made relating to the pharmacokinetics of medications. The future study includes the testing of the model in more varied clinical scenarios and the analysis of integration strategies using genomic data for a tailored approach. In order to expand the visibility and applicability of the present model and related claims, we plan on enhancing the verification of the model and implementing it in more sections of the health care system for the benefit of patients as well as their safety.

### Future work

Regarding future endeavors, there are several auspicious paths that need investigation. Firstly, by integrating various data sources, such as patient records and genomics data, we can enhance the capabilities of our model, resulting in more accurate and customized forecasts. Furthermore, the advancement of sophisticated explainable artificial intelligence (AI) methods might significantly improve the clarity of predictions by revealing the underlying decision-making process of the model. Furthermore, the implementation of our model as a real-time decision support system for healthcare workers has significant potential to enhance patient care by promptly flagging possible medication interactions during the provision of care. Moreover, the use of our model throughout the first phases of medication development provides pharmaceutical organizations with crucial discernments regarding chemical selection and anticipated adverse reactions. Engaging in partnerships with pharmaceutical companies for extensive clinical studies might authenticate the precision and practical significance of our methodology. Our model’s use for continuous medication safety monitoring serves as an advanced system that detects emergent drug interactions and adverse effects, hence improving patient safety through early warnings. These many research paths provide opportunities to fully use the capabilities of our model and transform the field of drug interaction prediction and management in healthcare and pharmaceutical research.

In order to create improvements in the integration of genomic data, the following pipelines will be employed: obtaining pipelines for the integration of genetic data into the GCN model; conducting studies with the enhanced model that combines genomic and pharmaceutical data; and examining the legitimacy of the improved model from a worldly point of view. Thus, our objective is to increase the quality of the prescribed therapies, better serving the patients and their safety, due to our partnership with genetic research centers.

To increase the verification of our Graph Convolutional Network (GCN) model, we propose to describe and perform many critical stages. We will perform thorough validation tests across diverse healthcare contexts, including different kinds of hospitals and clinics, to confirm the model’s resilience and generalizability. Collaborations with numerous healthcare institutes will enable us to collect varied datasets for extensive testing. Additionally, we want to incorporate the model into other areas of the healthcare system, such as outpatient services, inpatient care, and specialized treatment facilities, to assess its feasibility and usefulness in real-world circumstances. Developing user-friendly interfaces and decision assistance tools will simplify the model’s adoption by healthcare professionals, ensuring its greater application and boosting patient care.

## Data Availability

The available dataset online in link below: 10.6084/m9.figshare.7958747.v1.
